# Comparative Study for Efficacy and Safety of Adenoidectomy according to the Surgical Method: A Prospective Multicenter Study

**DOI:** 10.1371/journal.pone.0135304

**Published:** 2015-08-12

**Authors:** Jeong-Whun Kim, Hong Joong Kim, Woo Hyun Lee, Dong-Kyu Kim, Sung Wan Kim, Young Hyo Kim, Jung Gwon Nam, Seok-Won Park, Chan-Soon Park, Woo Yong Bae, Nam-Kyung Yeo, Tae-Bin Won, Seung Hoon Lee, Tae-Hoon Lee, Hyoung Joo Lee, Sang-Wook Kim, Sung-Wook Jeong, Jeong-Seok Choi, Doo Hee Han, Ji Ho Choi

**Affiliations:** 1 Department of Otorhinolaryngology, Seoul National University College of Medicine, Seoul National University Bundang Hospital, Seongnam, Korea; 2 Department of Otorhinolaryngology-Head and Neck Surgery, Bundang Jesaeng General Hospital, Daejin Medical Center, Seongnam, Korea; 3 Department of Otorhinolaryngology-Head and Neck Surgery, Chuncheon Sacred Heart Hospital, Hallym University College of Medicine, Chuncheon, Korea; 4 Department of Otorhinolaryngology-Head & Neck Surgery, Kyung Hee University, School of Medicine, Seoul, Korea; 5 Department of Otorhinolaryngology-Head and Neck Surgery, Inha University School of Medicine, Incheon, Korea; 6 Department of Otorhinolaryngology-Head and Neck Surgery, Ulsan University Hospital, University of Ulsan College of Medicine, Ulsan, Korea; 7 Department of Otorhinolaryngology-Head and Neck Surgery, Dongguk University College of Medicine, Ilsan Hospital, Goyang, Korea; 8 Department of Otorhinolaryngology-Head and Neck Surgery, St. Vincent's hospital, College of Medicine, The Catholic University of Korea, Suwon, Korea; 9 Department of Otorhinolaryngology-Head and Neck Surgery, College of Medicine, Dong-A University, Busan, Korea; 10 Department of Otorhinolaryngology, Gangneung Asan Hospital, University of Ulsan College of Medicine, Gangneung, Korea; 11 Department of Otorhinolaryngology, Seoul National University Hospital, Seoul National University College of Medicine, Seoul, Korea; 12 Department of Otorhinolaryngology-Head and Neck Surgery, Korea University Ansan Hospital, Korea University College of Medicine, Ansan, Korea; 13 Department of Otorhinolaryngology-Head and Neck Surgery, Busan St. Mary’s Medical Center, Busan, Korea; 14 Department of Otorhinolaryngology, Gyeongsang National University Hospital, Jinju, Korea; Affiliated Hospital of North Sichuan Medical College, CHINA

## Abstract

**Background/Objective:**

There have been several operative techniques for adenoidectomy and their efficacy and morbidity are different according to the technique. This prospective multicenter study was aimed to compare the efficacy and morbidity of coblation adenoidectomy (CA) with those of power-assisted adenoidectomy.

**Study Design:**

Prospective multi-institutional study.

**Methods:**

Children who underwent CA, power-assisted adenoidectomy with cauterization (PAA+C) or without cauterization (PAA-C) due to adenoid hypertrophy were enrolled from 13 hospitals between July 2013 and June 2014. Mean operation time, degree of intraoperative bleeding and postoperative bleeding rate were evaluated.

**Results:**

A total of 388 children (mean age ± standard deviation = 6.6 ± 2.5 years; 245 males and 143 females) were included. According to the adenoidectomy technique, the children were classified into 3 groups: (1) CA (n = 116); (2) PAA+C (n = 153); and (3) PAA-C (n = 119). Significant differences were not found in age and sex among three groups. In the CA group, mean operation time was significantly shorter (*P* < 0.001) and degree of intraoperative bleeding was significantly less (*P* < 0.001) compared to PAA+C or PAA-C group. Delayed postoperative bleeding rate of PAA-C group was significantly higher than that of CA or PAA+C group (*P* = 0.016).

**Conclusions:**

This prospective multicenter study showed that CA was superior to PAA in terms of mean operation time and degree of intraoperative bleeding.

## Introduction

The adenoids are a mass of lymphoid tissues located in the superoposterior area of the nasopharynx and affect breathing in the upper airway. It is known that, in general, the adenoids are tiny in size at birth and consistently grow during several years after birth due to the hyperactivity of the immune system.

Adenoid hypertrophy can lead to various symptoms such as nasal obstruction, mouth breathing, snoring, and speech abnormalities. It has also been known to be a risk factor for otitis media, dentofacial abnormality and obstructive sleep apnea syndrome [1]. Therefore, if the enlarged adenoids cause a variety of problems, surgical removal of the adenoids are generally required and adenoidectomy is one of the most commonly performed surgical treatment in the field of pediatric otorhinolaryngology [2].

There have been several operative techniques used for adenoidectomy. They include conventional curette adenoidectomy, monopolar suction diathermy adenoidectomy, power-assisted (or microdebrider) adenoidectomy (PAA), coblation adenoidectomy (CA), laser adenoidectomy and so forth [3–7].

There have been several comparative studies among these adenoidectomy techniques [8–13]. The coblation technique has advantages in that the surgical wand can work for ablation, coagulation, saline irrigation and suction at the same time [6]. Even if coblation adenoidectomy may shorten the time of surgery due to minimal intraoperative bleeding, to our knowledge, there was no study to compare coblation technique with power-assisted technique which is the most commonly used. Therefore, the objective of this prospective multicenter study was to compare the efficacy based on the operation time and morbidity based on intraoperative and postoperative hemorrhage between CA and PAA in children with adenoid hypertrophy.

## Material and Methods

### Subjects

We included children who (1) had various signs and/or symptoms related to adenoid vegetation such as nasal obstruction, mouth breathing, snoring and witnessed apnea; (2) were confirmed with adenoid hypertrophy based on a skull lateral radiograph, showing that the ratio of adenoid thickness to nasopharyngeal width (A/N ratio) was more than 25% (adenoid size was classified from 1 to 4 according to A/N ratio: grade 1, 0~25%; grade 2, 25~50%; grade 3, 50~75%; grade 4, 75~100%) [14]; (3) underwent CA or PAA between July 2013 and June 2014; and (4) completed postoperative follow up in 14 days after surgery. Recurrent infection was not indicated for surgery in the present study. We also excluded children who (1) had neuromuscular disorders or craniofacial anomalies; (2) previously underwent adenoidectomy; and (3) could not complete postoperative follow up. Surgeons who had performed more than 250 adenoidectomies participated in the study and the technique for adenoidectomy was chosen at the discretion of the surgeons. The present prospective and multicenter study was approved by the Institutional Review Board of each hospital (Seoul National University Bundang Hospital, Chuncheon Sacred Heart Hospital, Kyung Hee University Hospital, Inha University Hospital, Ulsan University Hospital, Dongguk University Hospital, St. Vincent's hospital, Dong-A University Hospital, Gangneung Asan Hospital, Seoul National University Hospital, Korea University Ansan Hospital, Busan St. Mary’s Medical Center, Gyeongsang National University Hospital). Written informed consent was obtained from the child’s parent and/or each child (7 years old or older) after a full explanation of the study.

### Surgical techniques

For CA, a coblator EVAC 70 wand (Arthrocare Co., Stockholm, Sweden) was used. Coblation is a controlled, non-heat driven, plasma-mediated radiofrequency-based ablation to dissolve enlarged adenoid tissues [6]. Under general anesthesia, CA was performed using an endoscopy or a microscope by an transoral approach.

For power-assisted adenoidectomy with cauterization (PAA+C), a microdebrider (Medtronic Xomed, Inc., Jacksonville, FL, USA) which is a powered rotary shaving device was used [5]. Under general anesthesia, adenoidectomy was carried out via a transoral or transnasal approach and then hemostasis was achieved by electrocauterization.

For power-assisted adenoidectomy without cauterization (PAA-C), a microdebrider was used as described above under general anesthesia and hemostasis was performed by packing with materials such as gauzes.

### Operation time for adenoidectomy

The operation time for adenoidectomy was defined as the period while the adenoidectomy was being performed. In addition, the time period spent for complete hemostasis of bleeding caused by adenoidectomy was also included.

### Amount of intraoperative bleeding

According to the category for assessment of intraoperative hemorrhage [15], intraoperative bleeding was classified from grade 0 to 5 (Table 1). In addition, these six grades were categorized into less amount of intraoperative hemorrhage (grades 0 and 1) and more amount of intraoperative hemorrhage (grades 2, 3, 4 and 5).

**Table 1:** Criteria for intraoperative bleeding.

Grade	Degree of bleeding	Suctioning required	Surgical field
0	no bleeding		
1	slight bleeding	no suctioning of blood required	
2	slight bleeding	occasional suctioning required	surgical field not threatened
3	slight bleeding	frequent suctioning required	bleeding threatens surgical field a few seconds after suction is removed
4	moderate bleeding	frequent suctioning required	bleeding threatens surgical field directly after suction is removed
5	severe bleeding	constant suctioning required	bleeding appears faster than can be removed by suction, surgical field is severely threatened and surgery is not possible

### Postoperative hemorrhage

All of the children were followed up and were evaluated for immediate (≤ first 24 hours after surgery) and delayed (> first 24 hours after surgery) postoperative bleeding for at least 2 weeks after surgery.

### Statistical analysis

The Statistical Package for the Social Sciences (SPSS) Version 20.0 (SPSS, Inc., Chicago, IL, USA) was used for statistical analysis. Continuous data are expressed as means ± standard deviation, and categorical data are presented as frequencies (percents). The one-way ANOVA test followed by a post hoc test (Bonferroni) was used to compare the mean age and adenoid size among CA, PAA+C, and PAA-C. The chi-square test was used to compare the sex and intraoperative bleeding among three procedures. The Kruskal-Wallis test followed by a post hoc test (Bonferroni) was used to compare the mean operation time among three methods. Binary logistic regression analyses were used to evaluate the risk for intraoperative bleeding of PAA+C or PAA-C, compared to CA. Fisher’s exact tests were used to compare postoperative bleeding among three operations. A statistical significance was considered to be reached at *P*-value < .05.

## Results

### Subjects

A total of 388 children (mean age = 6.6 ± 2.5 years; 245 males and 143 females), were included in the study (Table 2). They were classified into 3 groups based on the adenoidectomy techniques: (1) CA (n = 116); (2) PAA+C (n = 153); and (3) PAA-C (n = 119). There were no significant differences in age and sex among three groups. The adenoid size was smallest in the PAA+C group (*P* < 0.001).

**Table 2:** General characteristics of patients (N = 388).

		CA (n = 116)	PAA+C (n = 153)	PAA-C (n = 119)	*P* value
Age [yrs]		6.2 ± 2.5	6.6 ± 2.1	6.9 ± 2.8	0.108
Sex [n]					0.675
	Male	72 (62.1%)	94 (61.4%)	79 (66.4%)	
	Female	44 (37.9%)	59 (38.6%)	40 (33.6%)	
Adenoid size [grade]		3.0 ± 0.7^a^	2.5 ± 0.4^b^	2.9 ± 0.6^a^	<0.001*

Data are presented as mean ± standard deviation; CA, Coblation Adenoidectomy; PAA+C, Power-Assisted Adenoidectomy with Cauterization; PAA-C, Power-Assisted Adenoidectomy without Cauterization

*P value < 0.05; significant difference between a and b

### Operation time

**Fig 1:**
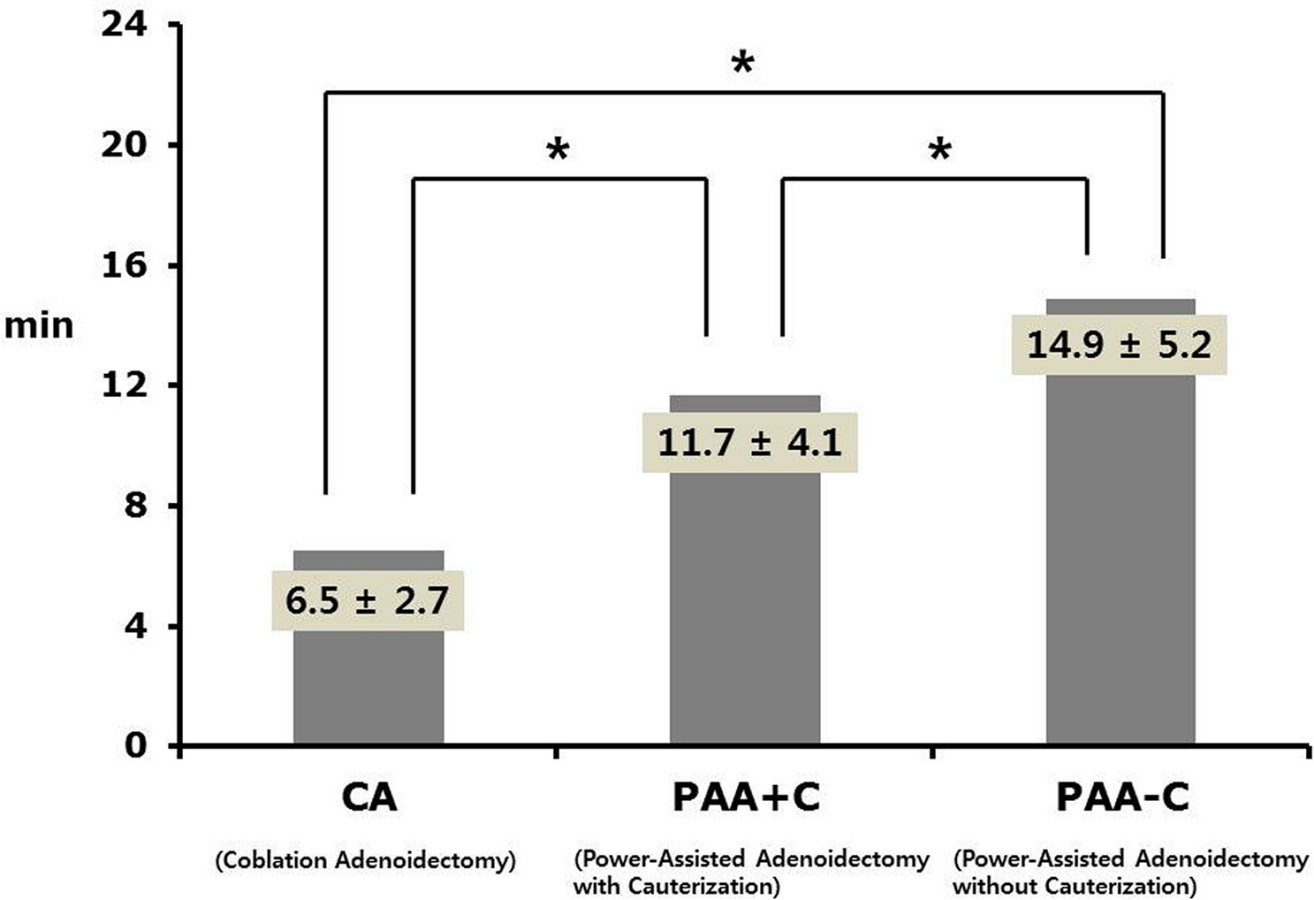
Comparison of mean operation time among CA, PAA+C, and PAA-C groups (N = 388).

The mean operation time of CA, PAA+C, and PAA-C was 6.5 ± 2.7, 11.7 ± 4.1, and 14.9 ± 5.2 minutes, respectively (*P* < 0.001). Post-hoc analyses indicated that significant differences were found in the mean operation times between CA and PAA+C (*P* < 0.001), CA and PAA-C (*P* < 0.001), and PAA+C and PAA-C (*P* < 0.001) (Fig 1).

### Intraoperative and postoperative bleeding

According to the category for assessment of intraoperative surgical field and hemorrhage, the proportion of less amount of intraoperative bleeding (grades 0 and 1) for CA, PAA+C, and PAA-C was 77.6%, 17.6%, and 5.0%, respectively. The proportion of grade 2 bleeding for CA, PAA+C, and PAA-C was 18.1% (21/116), 26.8% (41/153), and 50.4% (60/119), respectively. The proportion of grade 3 bleeding was 4.3% (5/116), 53.0% (81/153), and 38.7% (46/119), respectively. The proportion of grade 4 bleeding was 0%, 2.6% (4/153), and 5.9% (7/119), respectively. There was no grade 5 bleeding in all the three groups. There was a significant difference between the proportions of less and more amount of intraoperative bleeding across the three groups (*P* < 0.001) (Table 3). In reference to CA, PAA+C (*P* < 0.001) and PAA-C (*P* < 0.001) showed significantly higher risks of causing more amount of intraoperative hemorrhage after adjustment for age, sex, and adenoid size (Table 4).

**Table 3:** Comparison of intraoperative bleeding amount among CA, PAA+C, and PAA-C groups (N = 388).

	CA (n = 116)	PAA+C (n = 153)	PAA-C (n = 119)	*P* value
Less amount (Grades 0 and 1) [n]	90 (77.6%)	27 (17.6%)	6 (5.0%)	
				< 0.001*
More amount (Grades 2, 3, 4 and 5) [n]	26 (22.4%)	126 (82.4%)	113 (95.0%)	

*P value < 0.05

CA, Coblation Adenoidectomy; PAA+C, Power-Assisted Adenoidectomy with Cauterization; PAA-C, Power-Assisted Adenoidectomy without Cauterization

**Table 4:** The risk of causing more amount of intraoperative hemorrhage in PAA+C and PAA-C groups compared to the CA group.

	Odds Ratio	95% Confidence Interval	*P* value
CA	Reference		
PAA+C	20.134	10.184–39.805	< 0.001*
PAA-C	69.956	27.077–180.740	< 0.001*

*P value < 0.05

CA, Coblation Adenoidectomy; PAA+C, Power-Assisted Adenoidectomy with Cauterization; PAA-C, Power-Assisted Adenoidectomy without Cauterization

There was no immediate postoperative hemorrhage in three groups. However, delayed postoperative hemorrhage occurred in 4 patients after PAA-C. There was a significant difference in delayed postoperative bleeding among CA, PAA+C, and PAA-C (*P* = 0.016).

## Discussion

Even though the power-assisted technique is the most commonly used for adenoidectomy, use of the coblation technique is also increasing because it is known to be advantageous in that the device has multiple function including ablation, coagulation, suction and saline irrigation. However, its efficacy and morbidity has never been compared with the power-assisted technique for adenoidectomy. In order to compare the efficacy and morbidity between the two techniques, adenoidectomy-related procedures and intraoperative bleeding were investigated. The present prospective multicenter study showed that the coblation technique was superior to power-assisted technique for adenoidectomy in regard to the amount of intraoperative hemorrhage and time of operation. The shorter duration of procedure and less intraoperative bleeding with coblation adenoidectomy are associated with its basic principle of device operation. The coblation technique allows not only vaporization of the adenoid tissues but also blood coagulation at at the same time [6]. Therefore, the coblation technique does not require a separate coagulation process and provides a clear surgical field minimizing bleeding. However, since microdebrider does not have any function related with tissue coagulation, it needs a separate hemostatic process including electrical cauterization or compression by packing materials.

Conventional curette adenoidectomy, as one of the oldest adenoidectomy technique, has traditionally been performed to eliminate the enlarged adenoids. However, there were several weaknesses with this blind method including possibilities of incomplete removal and unintentional injury to adjacent structures [16]. In order to compensate these disadvantages, new instruments such as suction diathermy, microdebrider and coblator have been developed and used for adenoidectomy [4–6]. Furthermore, comparative studies have been carried out between conventional curette adenoidectomy and adenoidectomy using these new devices [8–11]. Many studies have showed that microdebrider or shaver adenoidectomy was better than conventional curette adenoidectomy in some respects [10,11]. A prospective randomized study compared transoral power-assisted endoscopic adenoidectomy (n = 26) with curettage adenoidectomy (n = 27) [10]. It showed that microdebrider adenoidectomy was superior in reduction of the adenoids and operation time compared to curettage adenoidectomy [10]. Another study also showed that partial adenoidectomy (removal of upper 1/2–3/4) using a microdebrider (n = 100) was quicker than partial adenoidectomy using a curette (n = 40) by 59% [11].

There have also been several studies demonstrating benefits of adenoidectomy using coblation [12,13]. A comparative study of 40 children showed that adenoidectomy using coblation was superior to adenoidectomy using cold curettage in pain and objective outcomes such as rhinomanometry and postoperative adenoid grade [12]. Another study also compared coblation technique with cold curettage in 60 children and it showed that coblation technique had significant advantages with regard to mean intraoperative blood loss and nasal mucociliary clearance rate [13]. To the best of our knowledge, the current study is the first prospective and multicenter trial to compare the coblation method with the microdebrider method. Although coblation adenoidectomy has advantages such as short operation time and low amount of intraoperative hemorrhage, it also has a disadvantage in cost due to higher price of coblation wands compared to microdebrider blades in Korea. Thus, when coblation adenoidectomy was selected among various surgical techniques, these advantages and disadvantages should be considered.

The degree of intraoperative bleeding was evaluated by category for assessment of intraoperative field in the present study. The degree of intraoperative hemorrhage can also be accurately measured by the volume of blood loss. However, it is difficult to measure the exact hemorrhage amount in case of coblation technique because bleeding is mostly very scanty. At last, we tried to apply the present surgical field method to estimate intraoperative bleeding amount and compare between groups in terms of surgical field. Intraoperative hemorrhage may be related with age, infection, adenoid size and so on. However, in the present study, there were no significant differences in age and sex among three groups. Recurrent infection was not indicated for surgery and postoperative wound infection was not found. In case of adenoid size, although the CA group showed larger adenoid size compared to PAA+C group, operation time and bleeding was more beneficial in the CA group. In the study, there were four cases with delayed postoperative bleeding after PAA-C, whereas there was no postoperative hemorrhage case after CA and PAA+C. All postoperative bleeding was minor and controlled by applying pressure using packing materials. No additional procedures were needed. We could not find any specific reasons for the minor bleeding such as fever or infection. According to a UK cohort study including 8,350 adenoidectomy subjects, the postoperative bleeding rates of curette adenoidectomy were 3.6 times higher than those of suction diathermy [17]. Considering these results, cauterization using diverse devices was thought to be more effective for bleeding control than cold dissection technique.

The strengths of our study are its prospective design and a relatively large sample size (388 pediatric patients from 13 hospitals). There were several limitations in the current study. Firstly, we did not compare the efficacy in terms of symptom improvement because we assumed that it may be confounded by tonsillectomy concurrently performed. In order to compare morbidity between the two techniques, we compared intra- and postoperative hemorrhage. However, postoperative pain was not compared because most of the surgeries are simultaneously performed with tonsillectomy and it may confound the analysis. Secondly, there may be a potential confounding factor associated with each surgeon’s surgical skill. However, to make up for this drawback, we tried to include skillful surgeons who had performed more than 250 adenoidectomies.

## Conclusion

Our prospective multicenter study demonstrated that coblation adenoidectomy was superior to microdebrider adenoidectomy with regard to time of operation and amount of intraoperative hemorrhage.
